# Differences in Larval Microhabitat Between Two Cryptic, Sympatric Salamander Species (*Desmognathus folkertsi* and *D. amphileucus*) in Northeastern Georgia, USA

**DOI:** 10.3390/ani15101479

**Published:** 2025-05-20

**Authors:** Carlos D. Camp, Erick M. Fortner

**Affiliations:** Department of Natural Sciences, Piedmont University, 1021 Central Avenue, Demorest, GA 30535, USA; efortner1111@lions.piedmont.edu

**Keywords:** coexistence, partitioning, niche, stream, riffle, flow rate, microhabitat, larvae, salamander, Appalachian Mountains

## Abstract

Sympatric species often exhibit habitat differences that promote coexistence. Two cryptic, sympatric salamander species, *Desmognathus folkertsi* and *D. amphileucus*, occupy streams across the Appalachian Mountains of northeastern Georgia, USA. Although frequently occurring together, the adults exhibit differences in microhabitat with *D. folkertsi* predominating in smaller streams and *D. amphileucus* more abundant in larger streams. Our purpose was to test for possible differences in larval habitat. Using a stream in which the two species coexist, we collected larvae from riffles, and biotic and abiotic variables associated with the stream and adjacent riparian zone were recorded. Because adults partially segregate by stream size, we noted the entry of a tributary that increased stream size and flow rate. Results showed a significant distribution of the two species upstream compared to downstream of the tributary, with no *D. amphileucus* found upstream; *D. folkertsi* was found throughout the study stream. We also found a significant difference between species in flow rate. We found no differences in any other variable that explained larval distribution patterns. We hypothesize that *D. folkertsi* nest in lower-flow riffles than *D. amphileucus*, resulting in the distribution of larvae, a pattern that may reflect selection on larval survival.

## Simple Summary

Adults of the cryptic, sympatric salamander species *Desmognathus folkertsi* and *D. amphileucus* are partially segregated by habitat, with *D. folkertsi* more abundant in small streams and *D. amphileucus* predominating in large streams. We investigated the larval distribution within a stream in northeastern Georgia, USA, in which the two species coexist, by examining several habitat variables. We found *D. folkertsi* larvae throughout the stream, but *D. amphileucus* were restricted to a reach downstream of a tributary. Because of the increased flow rate downstream and the significant difference between the two species in flow rate, we hypothesize that reproductive females respond to flow rate and choose nesting sites accordingly, a pattern which is reflected in the distribution of the larvae.

## 1. Introduction

The Competitive Exclusion Principle is a fundamental concept in ecology, stating that two species occupying the same ecological niche cannot coexist indefinitely [1]. Species that share similar ecological niches often coexist in sympatry because of habitat segregation, particularly when species are closely related phylogenetically and similar ecologically. This segregation has often been ascribed to resource partitioning resulting from competition [2,3,4,5,6,7]. However, habitat segregation or other factors enabling coexistence may have little or nothing to do with competition [8] and may include such things as predation [9,10,11], inherent preferences for different habitats [12,13,14,15,16,17,18,19], or an interaction among biotic and abiotic factors [20].

In amphibians, habitat segregation among larvae of anurans (e.g., [21,22,23]) and of pond-breeding salamanders (e.g., [24,25]) has been frequently studied. However, among the species-rich, plethodontid salamander communities of the Appalachian Mountains, relatively little research attention has been paid to habitat segregation among larval forms. This lack of attention is surprising given the intense research focus on habitat segregation that adult salamanders in these systems have received, research that has included both terrestrial [26,27,28] and stream-dwelling [28,29,30,31,32] species. Moreover, larval plethodontids frequently exist at high densities [33,34,35,36] and exert a large ecological influence on Appalachian stream ecosystems by impacting populations of aquatic prey [37,38] and potential competitors [39] as well as energy flow and nutrient cycling [40,41]. Larval salamanders are particularly important in headwater streams [40,41,42].

Salamanders, particularly those in the family Plethodontidae, have exhibited widespread cryptic speciation, resulting in phylogenetically related species that are extraordinarily similar in both morphology and ecology [43,44]. In stream-dwelling plethodontids, larval microhabitat preferences can help drive ecological differentiation and contribute to species coexistence. In one of the few studies of larval spatial occupancy in cryptic plethodontids, the larvae of sympatric two-lined salamanders (*Eurycea bislineata* species complex) segregate by habitat with one species (*E. wilderae*) dominating riffles and the other (*E. cirrigera*) found in pools [45].

The Dwarf Black-bellied Salamander (*Desmognathus folkertsi*) and the Southern Black-bellied Salamander (*D. amphileucus*, formerly *D. quadramaculatus* [46]) are two sympatric species occurring the southern Appalachian Mountains of northeastern Georgia, USA. These two species are cryptic in both their adult [47] and larval [48] morphology. Post-metamorphic individuals of these species partially segregate by habitat with *D. folkertsi* predominating along small streams, while *D. amphileucus* predominates in large streams [49]. However, little is known about how the larvae of these two species potentially segregate by habitat in stream ecosystems. Unlike the case of two-lined salamanders cited above [45], larvae of both *Desmognathus* species are largely restricted to riffles [35,50,51,52]. This habitat preference is perhaps a consequence of their tiny, white gills [53,54] and greater reliance on cutaneous respiration, which in turn requires turbulent water to prevent the buildup of a hypoxic boundary layer around the skin [55,56,57]. Darwin postulated that competition is often strongest among closely related species because of their similarity in form and ecology [58]; therefore, subtle differences in microhabitat preference may be a crucial mechanism facilitating coexistence between these larval forms.

In this study, we investigated the larval microhabitats of *D. folkertsi* and *D. amphileucus* within a stream in which the two species are abundant. Because of the differences in stream size exhibited by adults, we hypothesized that larvae of *D. folkertsi* and *D. amphileucus* exhibit distinct microhabitat differences, especially those related to stream size, which contribute to their ability to persist in sympatry.

## 2. Materials and Methods

**Figure 1:**
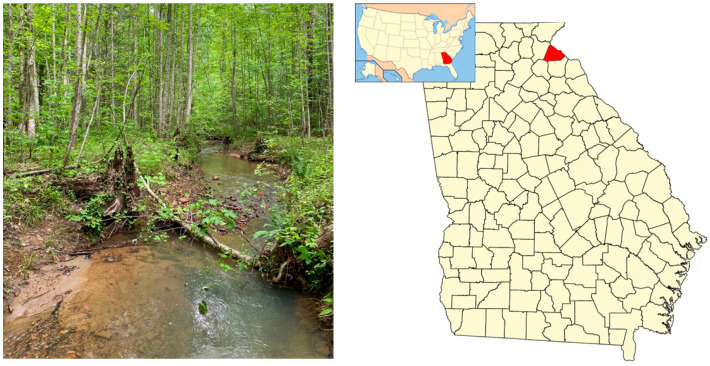
Stream used in investigation of habitat segregation between larval *Desmognathus amphileucus* and *D. folkertsi* located in Stephens County, Georgia; the state and county are highlighted on the respective USA and Georgia maps in red.

Our study stream was part of the Savannah River drainage and located in the Blue Ridge ecoregion [59] at an approximate elevation of 300 m in Stephens County, Georgia (34°37′58.8″ N; 83°19′51.6″ W; Figure 1). The stream is a small, 2nd order stream with a substrate that varies from scattered rock in riffle areas only 1–3 cm in depth to sandy runs of 2–5 cm in depth with some silt-bottom pools that approach 0.5 m deep. The deeper pools have submerged leaf packs. No aquatic vegetation is present. There are no waterfalls within the study reaches, although the stream passes over large areas of solid rock several hundred meters downstream of the study area.

**Figure 2:**
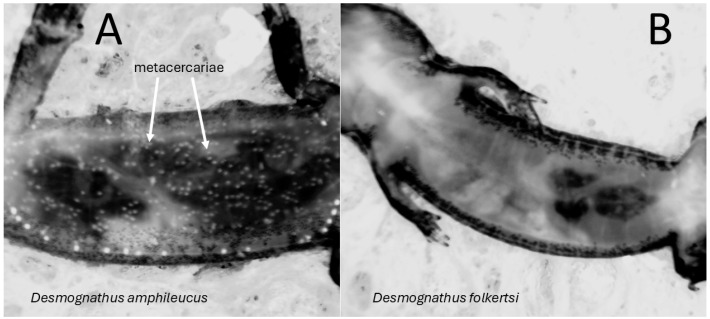
Ventral views of salamander larvae from study stream showing heavy infection of *Desmognathus amphileucus* by metacercariae of the trematode *Metagonimoides oregonensis* (**A**) and the absence of infection in *D. folkertsi* (**B**).

We chose this stream because the results of an earlier study, in which larvae of the two species were identified by PCR-based genotyping, demonstrated differential infection by metacercariae of the trematode *Metagonimoides oregonensis*. All of the *D. amphileucus* larvae were heavily infected, but none of the *D. folkertsi* were [60]. Because these trematodes encyst in the musculature [61,62], they are easily seen with magnification through the translucent ventral body wall of the larvae (Figure 2), giving a quick, convenient mechanism to identify these salamander larvae in the field. We acknowledge that our reliance on a single stream means that our conclusions, while informative of the interactions between the two species, may not apply across the full geographic range of overlap between them. However, we believe that the ease of larval identification in the field outweighed the cost of broader conclusions.

The stream is bordered by a flat, riparian zone ranging from 20 to 50 m in width (Figure 1). Steep hillsides form the boundaries of the riparian zone and at one point close onto the stream edge, forcing the water to flow through a small, narrow gorge. Because adults of the two salamander species partially segregate by stream size [49], we marked the entry of a tributary as a potentially significant landmark because of its obvious influence on the size and flow of the stream.

Unbroken, deciduous forest covers the riparian zone, the canopy being dominated by Sweetgum (*Liquidambar styraciflua*), Tulip Poplar (*Liriodendron tulipifera*), and Red Maple (*Acer rubrum*), with some American Beech (*Fagus grandifolia*). The understory is dominated by smaller American Beech, Silverbell (*Halesia tetraptera*), and American Holly (*Ilex opaca*). The surrounding slopes support a variety of oaks (*Quercus*). The slopes of the narrow gorge also contain stands of Great Laurel (*Rhododendron maximum*). Streamside herbaceous vegetation includes various grasses, ferns, and Doghobble (*Leucothoe fontanesiana*). The combination of mature, early successional species (e.g., Sweetgum and Tulip Poplar) and young old-growth species (e.g., American Beech) suggests a forest of moderate age along the riparian zone, while the oaks on the slopes indicate a much older forest [63,64,65].

We acquired salamander and stream data on three separate days in 2024 from 24 August to 10 September. The weather during this time was constant, with warm days and no rain. We chose riffles, i.e., where the surface was disturbed and tumbling as water flowed over shallow areas of loose rock, because this is the primary habitat of larvae of these species [35,50,51,52]. For very large riffles in which the water flowed at obviously different speeds, we treated the areas of different speed as different riffles. For each riffle, we measured the rate of water flow, width of streambed (as a permanent record of high-water flow), length of riffle, and mean diameter of substrate particles.

Prior to disturbing a riffle, we measured the flow rate by mixing powdered milk in the field, using enough powder to make the solution bright white. The time it takes a florescent dye to travel in a stream is a standard method of measuring flow rate [66,67]. However, florescent dyes can be toxic to aquatic life [68] even if they are considered “safe” because they do not pose an acute threat to human health [69]. Some authors have used food coloring as a harmless alternative to florescent dyes (e.g., [50]). We chose to use powdered milk because we could easily carry the amount needed as a powder on the hike (6 km) to the stream and mix the milk on site. We determined the time to the nearest 1/10 s for the milk to travel 1 m downstream through the riffle. Later, we took the reciprocal of each value to convert the raw data into a rate, i.e., m s^−1^.

To determine the mean particle diameter, we photographed each riffle prior to disturbance with a ruler placed in the center of the image. Later, using the imaging processing program Fiji (which runs on ImageJ-win32 [70]) in the lab, we arbitrarily chose 10 rocks within the image that were representative of the variation in rock size. We measured the diameter of each of these 10 rocks to the nearest mm, using the within-image ruler for calibration. We then determined the mean diameter for analysis.

Following these measurements, we disrupted each riffle with a net placed downstream, allowing the current to carry any salamander larvae into the net [71]. We slowly worked our way up through the entire riffle as earlier defined. If *Desmognathus* salamanders were found, we then measured streambed width and riffle length to the nearest 1/10 m. Following the complete search of each riffle, we identified individual salamanders, discarding the data for riffles in which no salamanders were found. To identify each salamander, we placed it in a plastic petri dish (85 mm in diameter) and immobilized the salamander with a wet sponge. Because of the strong righting reflex of these salamanders, this technique enabled the investigation of the belly while the specimen was upside-down (Figure 2) [60]. We then used a magnifying glass to determine whether the salamander had visible metacercariae (*D. amphileucus*) or not (*D. folkertsi*). Once all salamanders from a given riffle had been identified, we released them back into the riffle. To ensure that we were not missing *Desmognathus* salamanders in pool habitats, we thoroughly dip-netted 5–6 leaf packs in each reach and identified any salamanders present. We identified any two-lined salamanders as simply *Eurycea wilderae*/*cirrigera* because both species are present in the region, and the larvae are morphologically cryptic [45].

In addition to the variables associated with each riffle described above, we measured a series of environmental variables upstream and downstream of the tributary and determined if there were any meaningful differences between the two reaches. These variables included water temperature (°C), pH, dissolved oxygen (DO; mg/L), and mean water depth (cm). For the first three variables, we took two measurements for each reach. We used a pH meter (Henan Wanbang Ep Tech Co., Ltd., Shangqiu, China) to measure pH. For DO, we used a DO meter (Milwaukee Instruments^®^, Rocky Mount, NC, USA), adjusting the reading for temperature and elevation according to the manufacturer’s instructions. For the mean depth, we took 10 measurements of points representative of the full range of depths for each reach. We noted the frequency of pools, leaf packs, and coarse woody debris. We also visually compared the vegetation along both reaches.

We collected macroinvertebrates as potential salamander prey [52,72] from 7 to 8 riffles of each reach. For comparison of the two reaches, we generated a Jaccard Similarity Index [73], which ranges between 0, if there is no overlap in species, and 1, if both communities are identical in species content. To generate the Jaccard Index, we used an online calculator available at https://calculator.academy/similarity-index-calculator/ (accessed 30 April 2025). We also noted the frequency of pools, leaf packs, and coarse woody debris along with the presence of any fish, which are known predators of stream salamanders [74,75].

Prior to statistical analysis, we checked for potential violations of parametric assumptions, i.e., normality and homogeneity of variance. We normalized data as necessary with a log-transformation. We then used Pearson’s correlation of log-transformed variables to determine independence of those variables associated with riffles. For data that could not be normalized, we used a nonparametric (Spearman’s) correlation. For variables that were significantly correlated, we used the variable most likely to be detectable by salamanders for further analyses. Because riffle length is related to the amount of available habitat, we ran a Spearman’s correlation to test for an association between total salamander number per riffle and riffle length.

We then ran a generalized linear model (GLM) to test for the effect of mean particle size and flow rate on species presence in riffles. As part of the model, we included, as a covariate, the number of salamanders found in a riffle. We also ran a contingency analysis to test for departure from a random distribution of the two species between stream reaches, i.e., upstream and downstream of the tributary entry. We ran this analysis to determine whether the two species are distributed within the stream differentially relative to the two stream reaches. We ran a Mann–Whitney to test for a possible difference between *D. amphileucus* and *D. folkertsi* in the number of individuals found in riffles occupied by both species. We conducted this analysis to determine if one species predominates over the other, possibly as a result of competitive exclusion. We ran analyses in JASP ver. 19.1, a free statistical software that runs on the R platform [76].

## 3. Results

Measurements of temperature, pH, DO, and depth are given in Table 1. There was no appreciable difference between upstream and downstream reaches for any of these variables. Neither was there a meaningful difference in the vegetation of the stream banks or the riparian zone. Potential prey items found in riffles were dominated by stonefly larvae (Plecoptera) and small crayfish (Decapoda). We also found larval caddisflies (Ephemeroptera), true flies (Diptera), dragonflies (Odonata), and water penny beetles (Coleoptera). The two reaches were very similar in potential prey organisms, indicated by the relatively high value of Jaccard Similarity Index (0.76).

Leaf packs were associated with pools, which occurred at a frequency of 1–2 pools/50 m of stream length for both reaches. Coarse woody debris was present in both reaches in the form of fallen trees and limbs. Woody debris that partially interrupted stream flow was more abundant upstream. Small minnows of the family Cyprinidae were common in both reaches and were clearly able to swim freely between them.

Within leaf packs, we found larval two-lined salamanders (*Eurycea* sp.) in seven out of eight, downstream of the tributary. Two of the downstream leaf packs also had larval Red Salamanders (*Pseudotriton ruber*). Five of the seven upstream leaf packs contained larval two-lined salamanders. No *Desmognathus* larvae were found in any of the leaf packs.

In total, we searched 51 riffles, 44 of which had *Desmognathus* larvae. Downstream of the tributary, there were 32 riffles with 27 having salamanders, while 17 of the 19 riffles upstream were occupied by salamander larvae. The total salamander number per riffle ranged from 1 to 12 (mean = 2.25). The total number of salamanders per riffle was not significantly correlated with riffle length (Spearman’s rho = 0.237; *p* = 0.1206).

We found *D. amphileucus* in 20 riffles, all downstream of the tributary. The number per riffle ranged from one to six with a mean of 2.20. We found no *D. amphileucus* in any of the 19 riffles upstream of the tributary, although there were four collected in a riffle immediately (<10 m) downstream and there was a suitable riffle occupied by *D. folkertsi* within 10 m upstream of the tributary.

We found *D. folkertsi* in 33 riffles, 16 downstream and 17 upstream. The number of *D. folkertsi* per riffle averaged 2.125 (range = 1–8) downstream and 2.412 (range = 1–5) upstream. Fifteen of the downstream riffles yielded >1 salamander larvae, and nine of these contained individuals of both species. Larval *D. amphileucus* grows to be larger than larval *D. folkertsi*. Moreover, we also found several size classes of larvae of the same species occupying the same riffle. In addition to salamander larvae, we found seven adult female *D. folkertsi* attending nests upstream of the tributary.

Log-transformed data for flow rate, riffle length, and mean particle size were normally distributed, and Pearson’s correlation of these variables showed a significant association between flow rate and riffle length (Table 2). Spearman’s correlation with streambed width, which could not be normalized, showed a significant association with flow rate (Table 2). There was no other significant association between variables. Given the significant relationship between flow rate and both riffle length and streambed width, we chose flow rate for further analysis because we considered flow rate to be a variable most likely detectable by a salamander. Thus, the independent variables we used in our comparative analyses were mean particle diameter and flow rate.

**Figure 3:**
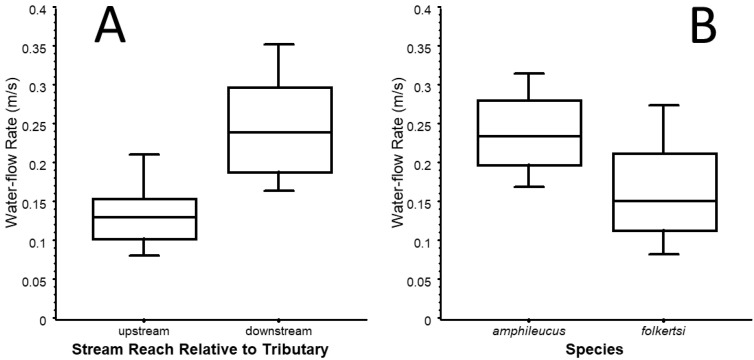
Comparisons of water-flow rates through stream riffles. (**A**) Comparison between stream reaches located downstream versus upstream of tributary entry. (**B**) Comparison between salamander species *D. amphileucus* and *D. folkertsi*. Whiskers represent, from bottom to top, 10% and 90% of data points; box edges represent 25% and 75%; midline represents median (50% of data points).

Riffles downstream of the tributary (N = 27) had a mean (±1 SE) particle diameter of 41.82 ± 2.62 mm, whereas those upstream had a mean of 41.94 ± 1.78 mm. Riffles downstream had a mean flow rate of 0.232 ± 0.10 m s^−1^. Those upstream had a mean flow rate of 0.129 ± 0.015 m s^−1^ (Figure 3A).

Riffles with *D. amphileucus* had a mean ± 1 SE particle diameter of 37.53 ± 0.67 mm, and riffles with *D. folkertsi* had a mean of 37.77 ± 0.39 mm. Riffles with *D. amphileucus* (N = 20) had a mean flow rate of 0.24 ± 0.01 m s^−1^, whereas riffles with *D. folkertsi* (N = 33) had a mean of 0.174 ± 0.014 m s^−1^ (Figure 3B). Results of the GLM indicated a significant effect on species presence (*p* = 0.018) with flow rate being the only significant variable (*p* = 0.005). The *p* value for each of the covariates of mean particle size and the number of salamanders in a riffle was 0.941.

Contingency analysis showed a significant departure from a random distribution between the two species relative to abundance upstream versus downstream of the tributary (χ^2^ = 14.444, df = 1, and *p* = 0.0001). The mean number of *D. amphileucus* within shared riffles was 2.333 ± 0.408. The mean number for *D. folkertsi* was 2.444 ± 0.729. The results of the Mann–Whitney used to test for differences in the number of individuals of each species within shared riffles was not significant (*p* = 0.6588).

## 4. Discussion

Our sampling confirmed that larvae of both species occupy riffles as their primary habitat. The fact that both species and multiple size classes frequently occurred together indicates that body size was not a major factor in determining species presence within a riffle, although size could conceivably influence the exact interstitial species that individuals occupy within the riffle. However, we did find different distribution patterns between the larvae of *D. folkertsi* and *D. amphileucus*. In our study stream, larval distribution of *D. folkertsi* and *D. amphileucus* were evidently determined by entry of a tributary, which increased both water flow and stream size. This was not an unexpected result given that *D. amphileucus* favors larger streams than does *D. folkertsi* [49]. However, we were surprised by the sharp boundary to the distribution of *D. amphileucus* larvae evidently set by the tributary.

We found only minor differences in most of the variables we measured between the upstream and downstream reaches. Temperature and pH were the same upstream and downstream. DO and depth exhibited small differences. In addition, the forest cover along the stream and associated terrestrial landscape was uninterrupted and similar throughout the lengths of the study reaches. Likewise, available prey items were also similar in both reaches. Stoneflies are particularly abundant in streams through late-successional deciduous forests of the region [77]. Therefore, the domination by stoneflies in our samples was not surprising.

Coarse woody debris that interfered with stream flow was more common upstream, probably as a result of the greater flow downstream during periodic events of heavy rain that can more easily push such obstacles aside. However, such woody debris was no more frequent than 2/50 m of stream and did not interrupt flow through riffles. While coarse woody debris can have a positive influence on aquatic life (e.g., [78,79]), it does not explain the absence of *D. amphileucus* upstream of the tributary.

The main difference between the upstream and downstream reaches of the tributary was water-flow rate, which in turn would influence the presence of coarse woody debris within the stream. Flow rate was also significantly different between riffles in which *D. folkertsi* versus *D. amphileucus* were found. Water-flow rate has been shown to play a critical role in structuring communities of aquatic life, both invertebrate [80,81,82,83] and vertebrate [84,85]. Moreover, flow rate would seem to be a variable that a salamander could easily detect.

We found no evidence that *D. amphileucus* is excluded from the upstream reach because of competition with *D. folkertsi*. For one thing, individuals of both species were found to coexist in the majority of riffles containing more than one salamander in the downstream reach. For another, larval *D. amphileucus* reach significantly larger sizes than larval *D. folkertsi* [47]. Moreover, *D. amphileucus* individuals are often aggressive towards other salamanders [86,87], and large larvae of the species have been observed preying on smaller ones in captivity [88]. Therefore, *D. amphileucus* would be more likely to exclude *D. folkertsi*, not the other way round.

It is entirely possible that the distribution of larvae is reflective of nesting patterns of the two species. Differences in stream habitat in sympatric, cryptic species of the two-lined salamander complex have been hypothesized to result from differences in site choice of nesting females [45]. Our observation of seven nesting *D. folkertsi* in the upstream reach suggests that reproductive females may actively select nesting sites based on flow rate. Both our study species have multi-year larval phases, and larvae tend to drift downstream over time [89]. Therefore, the absence of *D. amphileucus* upstream may be the result of females choosing higher-flow sites in which to nest, whereas some of the *D. folkertsi* larvae found downstream may have been the result of drift. Although water-flow rate evidently does not influence patterns of young deposition in a European salamander of the family Salamandridae (*Salamandra salamandra*) [90], the situation may be quite different for stream-dwelling plethodontids.

A preference by nesting *D. amphileucus* females to higher flow rates could be an adaptive strategy linked to their lengthy larval period of 3–4 years [71,91] compared to the 2-year larval period of *D. folkertsi* [92]. A longer larval period increases the risk of dehydration in smaller, more drought-prone sections of the stream, making high-flow areas more favorable for *D. amphileucus* larval survival. Although the southern Appalachians receive high levels of precipitation relative to surrounding regions [93], severe drought does occasionally occur, resulting in low stream flow [94,95,96,97] with severe drought causing even perennial portions of streams to dry [98]. In fishes, high juvenile survivorship associated with stable stream flows favors species with delayed maturity, whereas species that suffer high juvenile mortality caused by drought are characterized by early maturity [99,100]. Differences in hydrological patterns are known to influence survival in larvae of at least one species of stream-breeding plethodontid, i.e., *Gyrinophilus porphyriticus* [101,102], and may have consequences for other amphibian larvae as well [103].

The fact that our study involved a single stream means that our specific conclusions about larval distributions may not apply fully across the region of sympatry between *D. folkertsi* and *D. amphileucus*. However, the pattern of adult *D. folkertsi* predominating in small streams and *D. amphileucus* doing so in large streams is characteristic of multiple sites including completely different river systems [49]. Because the larvae are distributed similarly to adults, we feel that the pattern we observed in the larvae is likely, although not certain, to be widespread. Certainly, multiple factors can influence salamander abundance across the broader landscape (e.g., [104,105,106,107,108]). However, our data suggests that flow rate is a, if not the, major factor influencing within-stream distribution of our two study species relative to each other.

## 5. Conclusions

Our data showed a distribution of the larvae of two species along a study stream that parallels what has previously been reported for adults with *D. folkertsi* distributed farther up the smaller portion of the stream. The environmental variable that appeared to be important to this distribution was flow rate through the riffle habitat. Given that larval distributions likely align with adult reproductive-site selection, we hypothesize that adult female salamanders respond to variation in flow rates to choose nesting sites that optimize larval survival. Future investigators should look at different streams to determine if the pattern we observed is universal. In addition, they should examine whether female *D. folkertsi* and *D. amphileucus* actively select nesting sites based on flow rate or whether larvae exhibit behavioral responses to flow conditions. Additionally, long-term monitoring of larval survival across different flow regimes would provide insight into the adaptive significance of these habitat preferences.

## Figures and Tables

**Table 1:** Comparison of stream variables between reaches downstream and upstream of tributary entry. Absence of data under range means no variance in measurements.

	Downstream		Upstream	
	Mean	Range	Mean	Range
Temperature (°C)	15.0	---	15.0	---
pH	7.7	---	7.7	---
DO (mg/L)	7.05	7.0–7.1	7.3	
Depth (cm)	10.0	2.5–22.0	10.4	5.1–25.4

**Table 2:** Correlation analyses among measured stream variables. Numbers below the diagonal represent values for Pearson’s *r* except for correlations with streambed width, which are values for the nonparametric Spearman’s rho. The numbers above the diagonal represent corresponding *p* values with those marked with * being statistically significant.

	Flow Rate	Riffle Length	Mean Particle Diameter	Streambed Width
Flow rate	---	0.0292 *	0.8863	0.0012 *
Riffle length	−0.328	---	0.6404	0.2136
Mean particle diameter	0.022	0.073	---	0.8220
Streambed width	0.495	−0.190	−0.034	---

## Data Availability

Data will be made available upon request to C.D.C.
